# A Case of Neglected Bilateral Anterior Shoulder Dislocation: A Rare Entity with Unusual Mechanism of Injury

**DOI:** 10.1155/2015/461910

**Published:** 2015-04-20

**Authors:** Raghuram Choulapalle, Ramu Chokkarapu, Ravi Kanth Kolluri, Sreedhar Reddy Anne, Shanmuga Raju Perumal, Pavan Kumar Avadhanam, Ramesh Bheemanathuni

**Affiliations:** Department of Orthopaedics & Traumatology and Rehabilitation, Chalmeda Anand Rao Institute of Medical Sciences, Karimnagar, Telangana 505001, India

## Abstract

Bilateral shoulder dislocations are rare, and if they occurred, posterior type of dislocations is common. Bilateral anterior shoulder dislocations are very rare and occur due to trauma with unique mechanism of injury. We report a case of unreduced simultaneous bilateral anterior dislocations of shoulder without associated fractures in a forty-year-old man following a unique mechanism of injury; both hands of the patient were pulled from either side. To the best of our knowledge, this unusual mechanism of injury has not been reported in the literature.

## 1. Introduction

Anterior glenohumeral dislocations are most commonly encountered major joint dislocations in emergency room. Bilateral shoulder dislocations with or without fractures are rare. For a simultaneous bilateral dislocation to occur, the forces must act synchronously on both shoulders in a similar manner. Bilateral posterior dislocations are commonly seen during electrocution, convulsions, or hypoglycemic seizures due to violent contractions of internal rotators of shoulder. There are few reported cases of bilateral anterior shoulder dislocations with different mechanisms of injury: at the start of backstroke swimming competition, bench pressing athlete, fall on elbows, postseizure episode, trying to prevent a backward fall by extending both arms behind the back, and, in our case, pulling of both hands from either side with both limbs in abduction and external rotation.

## 2. Case Report

**Figure 1:**
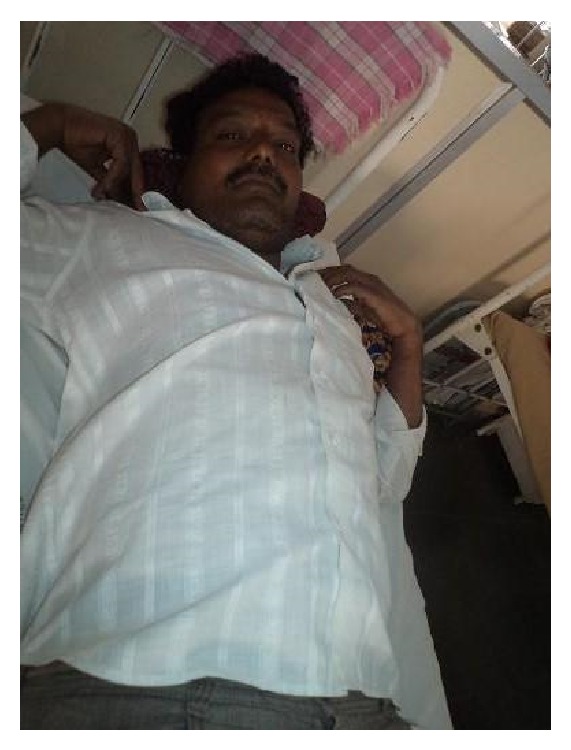
Patient with bilateral anterior shoulder dislocation.

A forty-year-old man presented to our hospital in December 2013 with pain and restricted range of movements in both shoulders. Patient was involved in a quarrel six weeks back and both the hands of the patient were held by two people on either side and were pulled. He had no history of seizures, previous shoulder dislocations, or laxity of other joints. On examination, arms were abducted and externally rotated. There was loss of round contour of shoulder with fullness over anterior aspect of both shoulders. No neurovascular injury and no associated fractures were confirmed by radiographs and CT scan in both arms (Figures 1 and 3). Patient was able to perform flexion up to 30 degrees and abduction and external rotation with no pain restraint. Adduction and internal rotations were restricted (Figure 1).

**Figure 2:**
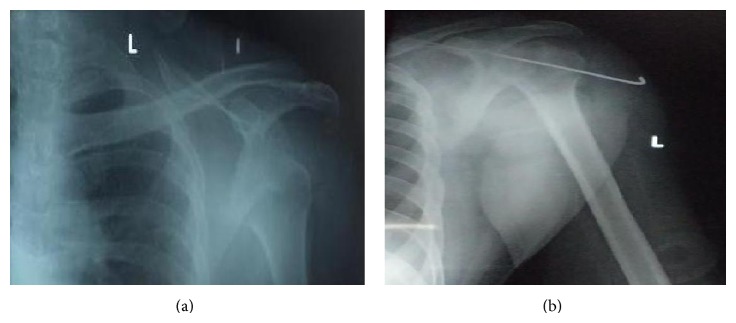
Radiographs of left anterior dislocation of shoulder (preoperative and postoperative).

**Figure 3:**
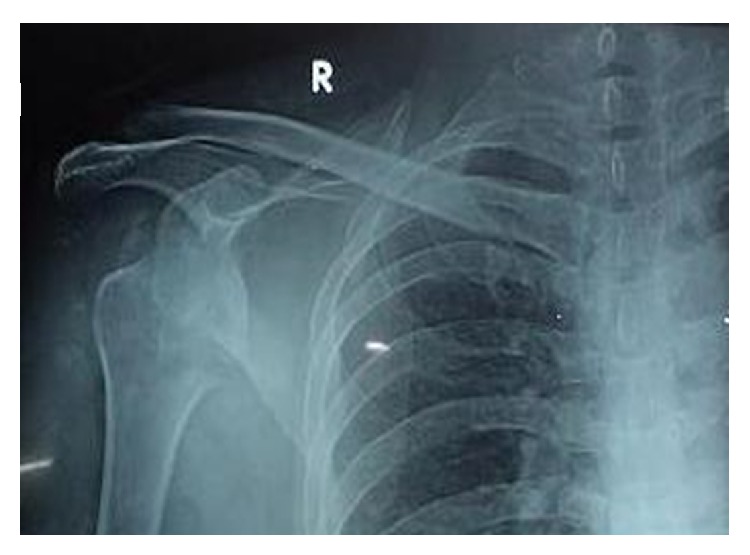
Radiographs of right anterior shoulder dislocation (preoperative).

Old unreduced dislocations are difficult to reduce by closed methods because of soft tissue contractures, fibrous tissue in the glenoid cavity, retracted rotator cuff muscle, iatrogenic fractures, and neurovascular damage. Left shoulder was operated on six weeks back. Soft tissue release and open reduction of the head into glenoid cavity was done through deltopectoral approach and secured with k-wires (Figure 2).

## 3. Discussion

Unilateral anterior shoulder dislocations account for 95% of all shoulder dislocations, but simultaneous bilateral anterior dislocations are a clinical rarity because almost always one extremity takes the brunt of impact during trauma incidence [1]. Axial loading with arm in internal rotation and adduction produces posterior dislocation, which is common in electrical shock or postseizure episode due to violent contraction of internal rotators of shoulder. Mechanism of bilateral anterior dislocation is combination of forced extension, abduction, and external rotation of both arms and these forces need to be symmetrical and simultaneous. Acute bilateral anterior dislocations have to be reduced quickly with various closed methods: the Stimson gravity method, Kocher maneuver, or Milch maneuver. Unreduced bilateral anterior dislocations cannot be reduced by closed methods because of fibrous tissue in glenoid cavity, risk of iatrogenic fractures, soft tissue interposition, and neurovascular damage.

Simultaneous bilateral anterior shoulder dislocations are rare. They have a unique mechanism of injury and were first described in 1902 in patients in whom excessive muscular contraction occurred as a result of camphor overdose by Mynter [2]. Cresswell in 1998 reported a case of bilateral anterior dislocation of the shoulder without any fractures in a bench pressing athlete [3]. Lasanianos and Mouzopoulos in 2008 reported a case of bilateral anterior dislocation of the shoulders after a seizure episode [4]. Turhan and Demirel in 2008 reported a bilateral anterior dislocation case in a horse rider [5], Felderman et al. in 2009 reported a similar case in a 44-year-old woman who was doing chin-up exercise [6], Thakur et al. reported a similar case where patient was trying to prevent a backward fall by extending both arms behind his back [7], Dlimi et al. in 2012 reported a case of bilateral anterior dislocation of the shoulders at the start of a backstroke swimming competition [8], and Yashwantha et al. in 2013 reported a similar case due to a fall over pointed elbows [9]. In our case, it was pulling of both hands on either side, with both arms in abduction and external rotation, which is a unique mechanism of injury never described in the literature (Table 1).

## Figures and Tables

**Table 1:** Different mechanism of injury in bilateral, simultaneous anterior shoulder dislocation.

Author	Journal	Year	Associated fractures	Systemic diseases	Mechanism of injury
Cresswell [3]	British Journal	1998	Nil	Nil	Bench pressing

Jarvela and Salmela [1]	SICOT online report E028	2003	Humeral neck fracture and rotator cuff tear on left side	Nil	Convulsions form hypoglycemic shock

Devalia and Peter [10]	J Postgrad Med	2005	Greater tuberosity fracture right side	Nil	Landing on either side of ladder on his out stretched hands

lasanianos and Mouzopoulos [4]	Cases Journal	2008	Greater tuberosity fracture and Hill-Sachs lesion	Seizure disorder	Violent muscle contraction

Turhan and Demirel [5]	Arch Orthop Trauma Surg	2008	Nil	Nil	Horse riding

Abalo et al. [11]	E. pub	2008	Nil	Nil	Fall

Felderman et al. [6]	Journal of Emergency Medicine	2009	Nil	Nil	Chin-up exercises

Thakur et al. [7]	Journal of Clinical and Diagnostic Research	2010	Nil	Nil	Backward fall by extending both arms behind his back

Mofidi et al. [12]	American Journal of Emergency Medicine	2010	Temporomandibular dislocation	Generalized tonic-clonic seizure	Violent muscle contraction

Silva et al. [13]	Rev Bras Ortop	2011	Nil	Nil	Posterior fall

Dlimi et al. [8]	J Orthop Traumatol	2012	Nil	Nil	Backstroke swimming competition

Yashwantha et al. [9]	Journal of Orthopaedic Case Reports	2013	Nil	Nil	Fall on pointed elbows

Our case			Nil	Nil	Pulling of both hands on either side
